# Potential of ferroptosis and ferritinophagy in migraine pathogenesis

**DOI:** 10.3389/fnmol.2024.1427815

**Published:** 2024-06-10

**Authors:** Michal Fila, Lukasz Przyslo, Marcin Derwich, Jolanta Luniewska-Bury, Elzbieta Pawlowska, Janusz Blasiak

**Affiliations:** ^1^Department of Developmental Neurology and Epileptology, Polish Mother’s Memorial Hospital Research Institute, Lodz, Poland; ^2^Department of Developmental Dentistry, Medical University of Lodz, Lodz, Poland; ^3^Faculty of Medicine, Collegium Medicum, Mazovian Academy in Plock, Plock, Poland

**Keywords:** migraine, iron, brain iron deposition, ferroptosis, ferritinophagy, migraine chronification, periaqueductal gray

## Abstract

**Objective:**

To assess the potential of ferroptosis and ferritinophagy in migraine pathogenesis.

**Background:**

Ferroptosis and ferritinophagy are related to increased cellular iron concentration and have been associated with the pathogenesis of several neurological disorders, but their potential in migraine pathogenesis has not been explored. Increased iron deposits in some deep brain areas, mainly periaqueductal gray (PAG), are reported in migraine and they have been associated with the disease severity and chronification as well as poor response to antimigraine drugs.

**Results:**

Iron deposits may interfere with antinociceptive signaling in the neuronal network in the brain areas affected by migraine, but their mechanistic role is unclear. Independently of the location, increased iron concentration may be related to ferroptosis and ferritinophagy in the cell. Therefore, both phenomena may be related to increased iron deposits in migraine. It is unclear whether these deposits are the reason, consequence, or just a correlate of migraine. Still, due to migraine-related elevated levels of iron, which is a prerequisite of ferroptosis and ferritinophagy, the potential of both phenomena in migraine should be explored. If the iron deposits matter in migraine pathogenesis, they should be mechanically linked with the clinical picture of the disease. As iron is an exogenous essential trace element, it is provided to the human body solely with diet or supplements. Therefore, exploring the role of iron in migraine pathogenesis may help to determine the potential role of iron-rich/poor dietary products as migraine triggers or relievers.

**Conclusion:**

Ferroptosis and ferritinophagy may be related to migraine pathogenesis through iron deposits in the deep areas of the brain.

## 1 Introduction

Aberrant iron metabolism is reported in many conditions, including neurological disorders, in particular stroke and neurodegenerative diseases (Long et al., 2023). Headaches may be also associated with disruption of iron metabolism, which is demonstrated by a positive correlation between iron deficiency anemia and chronic daily headache (Singh et al., 2023). Iron can be important in migraine, due to enhanced oxidative stress in the migraine-affected brain and the potential of iron ions to catalyze the Fenton and Haber-Weiss reactions to produce reactive oxygen and nitrogen species (RONS) that further increase oxidative stress in the brain (Borkum, 2021). Also, although migraine is a major cause of disability, fifth in the general population and second in young women, the mechanisms of its pathogenesis are poorly known, making migraine an undiagnosed and undertreated disease (Gupta and Gaurkar, 2022). Therefore, studies on molecular mechanisms of migraine pathogenesis are justified, especially since access to the human target material is restricted and animal models of human migraine have serious limitations (Harriott et al., 2019).

There is not an equivocal relationship between dietary intake of iron and migraine prevalence or severity. A large cohort study demonstrated that dietary iron intake might be differentially related to migraine and severe headaches in dependence on sex and age (Meng et al., 2021). Several studies report an association between iron deficiency anemia and migraine, but there are many concerns about those studies. Therefore, it is important to explore mechanisms behind the role of iron homeostasis in migraine pathogenesis to determine whether iron-rich dietary products can be migraine triggers or relievers.

Iron imbalance and resulting oxidative stress and lipid peroxidation may lead to ferroptosis, a kind of programmed cellular death that may be related to ferritinophagy, a class of specific autophagy. Ferroptosis plays a role in the pathogenesis of cancer, inflammation, neurodegenerative diseases, stroke, and other CNS disorders (Jin et al., 2024). Although migraine is not a typical neurodegenerative disease, a loss of neurons induced by pyroptosis, another kind of programmed cellular death, was observed in a mouse model of human migraine (Wang et al., 2022).

In this narrative/hypothesis mini review, we present some information on iron metabolism in the brain and the association of migraine with iron deposits in various brain areas as well as the role of ferritinophagy and ferroptosis in iron metabolism and their potential in migraine pathogenesis.

## 2 Iron homeostasis in the brain

In the central nervous system (CNS), iron is essential for many processes, including DNA synthesis, myelination of neurons, mitochondrial quality control, gene expression, synthesis, and metabolism of catecholamine neurotransmitters (Cheli et al., 2020). The most remarkable aspect of aberrant iron metabolism in CNS is its role in the pathogenesis of Alzheimer disease, Parkinson disease, Fridrich ataxia, sporadic Creutzfeldt-Jakob disease, Hallervorden-Spatz syndrome, and other neurological symptoms (Piñero and Connor, 2000).

Iron can participate in the electron-transfer reaction, which is important for its homeostasis, but also the main reason for the toxicity of free iron. Most of the iron in circulation is absorbed by serum transferrin (Tf). Tf-bound iron is taken by most parenchymal cells through the transferrin receptor protein 1/2 (TfR1/2) (Klausner et al., 1983). Iron that is not bound by Tf is taken up by the divalent metal transporter 1 (DMT1) and Zip14/Slc39a14, a member of the SLC39A zinc transporter family (Liuzzi et al., 2006). Within cells, iron is associated with cytosolic ferritin (FT).

To reach the brain, systemic iron must cross BBB or brain-cerebrospinal fluid barrier. BBB comprises brain microvascular endothelial cells (BMVECs) supported by astrocytes, neurons, and pericytes (Abbott et al., 2006). Iron can cross BBB as free iron ions or transferrin-bound iron (TBI). Ferric (Fe(III)) iron is bound to the transferrin receptor forming the holo-Tf complex. Ferrous (Fe(II)) iron can enter BMVECs directly through the solute carrier family 39 member 8 (SLC39A8, ZIP8) or solute carrier family 39 member 14 (SLC39A14, ZIP14) divalent ion transporters. In the TBI uptake pathway, iron is bound to Tf protein, which is then bound to TfR1 forming holo-Tf-TfR1 complex, which can be endocytosed. Iron is reduced in the endolysosome to Fe(II) and released into the cytosol with the involvement of DMT1 (Rouault, 2013). In the non-transferrin-bound iron (NTBI) uptake pathway, extracellular free ferric ions Fe(III) or TBIs are reduced by the metalloreductases STEAP2 and STEAP3 (Kosman, 2020). In the BMVEC cytoplasm of the BMVEC, ferrous iron can be bound by the PCBP1 and PCB2 iron chaperones for storage in FT, included in cytosolic iron-dependent enzymes, or transported to the mitochondria for assembly of the F-S clusters and heme. Iron accumulated in BMVEC can be apically exported into circulation or basolaterally into the abluminal space. If needed, FT is degraded in lysosomes, and ferrous ions are released into the cytosol with the involvement of the STEAP2 and STEAP3 and lysosomal membrane ascorbate-dependent ferrireductase CYB561A3 (Lcytb) (Meng et al., 2022). Solute carrier family 40 member 1 (ferroportin, FPN, FPN1) is the key protein facilitating ferrous iron efflux (Pan et al., 2020). It requires the oxidation of ferrous ions (ferrioxidation) by a ferroxidase, ceruloplasmin (CP), or hephaestin (HP) (McCarthy and Kosman, 2015).

When the FT system and mechanisms involved in iron export fail, its concentration in the brain increases, which may lead to its accumulation and pathological consequences. In neurons, iron accumulation may result in different forms of programmed cell death, including ferroptosis and paranthatos, and aberrant autophagy, which may also lead to cell death, contributing to neurodegeneration (Fang et al., 2018). Iron overload in glial cells may result in inflammation also promoting neurodegeneration (Levi et al., 2024).

The role of iron in migraine pathogenesis may be underlined by its dietary intake, but there are surprisingly few studies on the association between dietary iron and migraine. The development of high-resolution neuroimaging resulted in the discovery of the deposits of increased iron in some deep areas of the brain of migraine patients.

## 3 Migraine-related iron deposits in the brain

The periaqueductal gray matter (PAG) is a brain structure important in the propagation and modulation of pain and was recently shown to be involved in migraine attacks and chronification (Cao et al., 1999; Bahra et al., 2001; Vilas et al., 2023). An early study investigated iron homeostasis in patients with episodic migraine (EM) or chronic daily headache (CDH) between attacks and patients with CDH during headache with high-resolution magnetic resonance imaging (MRI) in PAG, red nucleus (RN), and substantia nigra (SN) (Welch et al., 2001). PAG might be a source of migraine attacks, likely due to a dysfunctional control of the trigeminovascular nociceptive system. Therefore, that study showed an increased iron deposition in PAG of migraine and headache patients that might disturb the central antinociceptive neuronal network. The continuing question is “How?”

Kruit et al. assessed iron concentration in various areas of the brain by MRI measures in a large population-based cohort of migraine patients and controls that were stratified into two groups – younger than and older than or equal to 50 years (Kruit et al., 2009). The population of patients was derived from the Dutch population-based Genetic Epidemiology of Migraine (GEM) study and the Cerebral Abnormalities in Migraine, an Epidemiological Risk Analysis (CAMERA) MRI Study (Launer et al., 1999; Kruit et al., 2010). The authors concluded that repeated migraine attacks might increase iron concentration in multiple deep nuclei that are involved in central pain processing and migraine pathogenesis, but the potential causative role of that iron accumulation in migraine required further research.

Another MRI study aimed to assess the differences in the deposition of iron in the brain between migraine/headache patients and controls concerning disorder type, attack frequency, and illness duration (Tepper et al., 2012). The authors showed different MRI measures in the bilateral GP of all patients with migraine, patients with EM, and patients with CDH.

In a CAMERA follow-up study the MRI measures of the putamen, posterior putamen, nucleus caudates, and substantia nigra pars compacta were evaluated in migraine patients who had participated in the original CAMERA project 9 years earlier (Palm-Meinders et al., 2017). No significant differences were observed between migraine patients and controls. The differences observed earlier were not detected in that study and the authors did not present a convincing hypothesis explaining the time course of the changes they had observed.

After almost two decades of reports on increased iron deposits in the brain of migraine patients, Dominguez et al. showed that patients with CM displayed larger iron ground volume in RN and larger iron deposits in PAG as compared to patients with EM (Domínguez et al., 2019). Such differences in PAG were also seen between EM patients and controls. No significant differences were observed in GP. A positive correlation was observed between iron grounds volume in PAG with plasma levels of soluble tumor necrosis factor-like (TNF) weak inducer of apoptosis (TWEAK), a marker of vascular inflammation, and cellular fibronectin. Therefore, iron grounds volume in RN and iron deposits in PAG may differentiate between patients with episodic and chronic migraine and consequently add a biomarker to the differential diagnosis of migraine variants. Given that PAG is activated during migraine attacks the authors hypothesized that repeated migraine attacks and PAG activation could increase oxidative stress, overproduction of RONS, and consequently cell damage associated with hyperemia and iron deposition.

An MRI-based longitudinal study determined the association between iron deposition in the brain of migraine patients and their response to erenumab, a drug that blocks calcitonin gene-related peptide (CGRP) receptors in fibers and ganglia of the trigeminal system (Nikolova et al., 2023). The difference between erenumab responders and non-responders was seen after 8 weeks of treatment suggesting that the lesser iron brain accumulation, the better response to erenumab.

The results of Dominguez et al. were largely confirmed in a recent study with 200 migraine patients (Xu et al., 2023). That study reported an increased iron deposition in the putamen, caudate, and nucleus accumbens (NAC) in migraine patients as compared to controls. Patients with CM had a higher volume of iron deposits compared to EM in multiple subcortical nuclei, especially in NAC, and those deposits were postulated to be useful for the differential diagnosis of EM and CM. Higher iron deposition in NAC was associated with disease progression. A recent study reported an increased iron deposition in the extrapyramidal system in episodic migraine (Li et al., 2024).

In summary, chronic migraine patients may have increased iron deposits in PAG as compared to patients with episodic migraines, who, in turn, may have more iron deposition in PAG than controls. Iron brain deposition in PAG may disturb processes controlling the antinociceptive network. However, the study of Dominguez et al. elicited some criticism. One of the main objectives against the conception that migraine is causatively associated with iron deposits is increased iron deposition in the brain of the elderly (Li G. et al., 2023). The prevalence of migraine decreases in older age along with the number of attacks and their intensity. However, we presented arguments that the age-dependence of migraine prevalence may reflect two aspects: inborn or acquired susceptibility to migraine and stress load during lifetime (Fila et al., 2023a). If iron belongs to these inborn/acquired susceptibility factors, decreased stress in older age might compensate for increased brain iron deposits.

## 4 Ferroptosis, ferritinophagy and their potential in migraine

Free Fe(II) ions may catalyze the Fenton and Haber-Weiss reactions resulting in an increased production of RONS that may damage brain cells. In extreme cases, cellular damage may lead to spontaneous or programmed cell death. Migraine is not a neurodegenerative disease, and its pathogenesis is not featured by massive cell death. However, apoptosis and pyroptosis, programmed cell deaths, are reported in migraine (Wang et al., 2022).

Elevated RONS may induce a wide spectrum of damages to cellular biomolecules, including lipid peroxidation, a process that was reported in migraine patients (Tuncel et al., 2008). Accumulation of iron in the brain may increase oxidative stress, induce lipid peroxidation, and trigger ferroptosis, a kind of programmed cellular death that plays a role in the pathogenesis of neurodegenerative diseases, stroke, and other CNS disorders, its implications in inflammation and metabolism suggest its potential in migraine pathogenesis (Li and Huang, 2022; Long et al., 2023; Jin et al., 2024).

**Figure 1:**
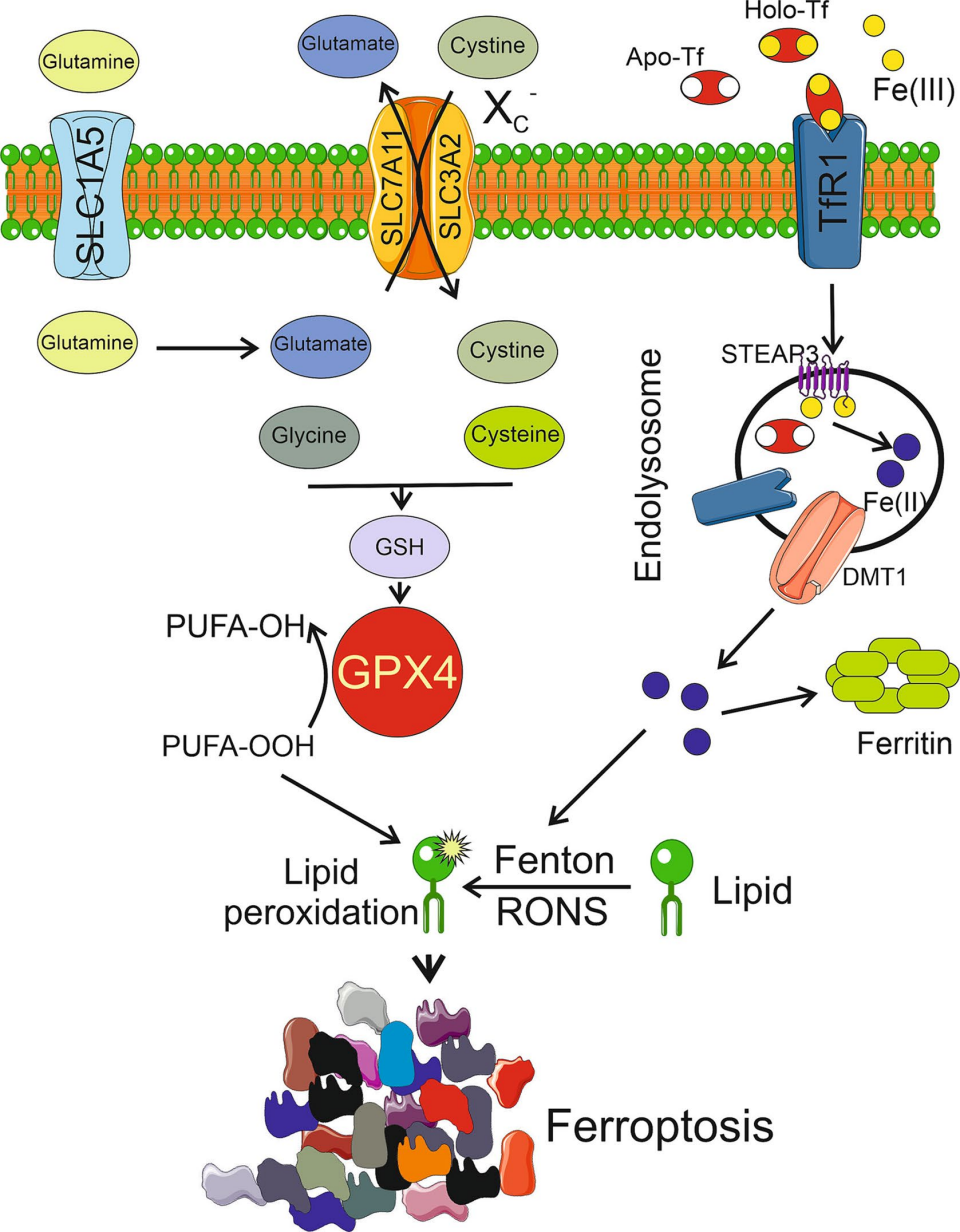
Ferroptosis is activated by excessive lipid peroxidation caused by reactive oxygen and nitrogen species (RONS) generated in the iron-mediated Fenton and Haber-Weiss reactions (Fenton). Lipid peroxidation is also generated by an impaired antioxidant system, mainly glutathione peroxidase 4 (GPX4), which reduces polyunsaturated fatty acids peroxides (PUFA-OOH) into alcohols (PUFA-OH). Reduced glutathione (GSH), a powerful antioxidant, is a cofactor of GPX4 synthesized from cysteine and glutamate whose levels are regulated by the X_C_^−^ system consisting of light chain, solute carrier family 7 member 11 (SLC7A11), and heavy chain, solute carrier family 3 member 2 (SLC3A2). Glutamate is produced from glutamine transported to the cell by solute carrier family 1 member 5 (SLC1A5). Extracellular Fe(III) ions are bound by apo-transferrin (Apo-Tf) to form holo-transferrin (Holo-Tf) that is bound by transferrin receptor 1 (TfR1) and reduced to Fe(II) by metalloreductase STEAP3 after endosomal uptake and then released by divalent metal transporter 1 (DMT1). Several other objects, including enzymes, and other pathways of ferroptosis are not presented in this scheme for clarity. Other pathways of ferroptosis are possible. Parts of this figure were drawn by using pictures from Servier Medical Art. Servier Medical Art by Servier is licensed under a Creative Commons Attribution 3.0 Unported License (https://creativecommons.org/licenses/by/3.0/).

Iron-mediated and RONS-induced lipid peroxidation is essential for the initiation of ferroptosis (Figure 1). This, in turn, suggests the involvement of the glutathione (GSH) peroxidase 4 (GPX4) cellular antioxidant system, which is the only cellular glutathione peroxidase to reduce lipid peroxides into lipid alcohols. GPX4 is assisted by the X_C_^−^ system, which is a heterodimeric antiporter of Cys and Glu consisting of light chain, solute carrier family 7 member 11 (SLC7A11), and heavy chain, solute carrier family 3 member 2 (SLC3A2) (Lewerenz et al., 2013; Li et al., 2022). The X_C_^−^ system mediates the influx of cystine and outflux of Glu. Inside the cell, cystine is reduced by GSH or thioredoxin reductase 1 (TRXR1) to Cys, which is then used for GSH biosynthesis (Lewerenz et al., 2013). Iron-mediated lipid peroxidation results in the oxidation of polyunsaturated fatty acids (PUFAs) in the membrane lipids, which is facilitated by an impaired antioxidant system. Ferroptosis is stimulated by Acyl-CoA synthetase long-chain family member 4 (ACSL4), which specifically catalyzes the synthesis of PUFAs, including arachidonoyl and adrenoyl acids that are crucial for supplying cellular membrane with PUFAs (Kagan et al., 2017). Several mechanisms have been suggested to underlie ferroptosis-related PUFA oxidation after their synthesis mediated by ACSL4, involving nicotinamide adenine dinucleotide phosphate oxidases, lipoxygenases, cytochrome P450 oxidoreductase coupled to cytochrome P450 monooxygenases, and RONS produced by the impaired mitochondrial electron transport chain (Chen et al., 2020).

Excessive heme and non-heme iron can directly induce ferroptosis, which can be blocked *in vivo* and *in vitro* by iron chelators and supplementation with iron increases the cell susceptibility to ferroptosis, and lipid peroxidation, a mediator of ferroptosis, can be induced by iron-stimulated RONS production. Therefore, it can be hypothesized that increased iron deposits in certain structures of the brain of migraine patients can mediate ferroptosis in these structures. However, it is not easy to answer the question of how ferroptosis can be related to migraine, because the origin and consequences of increased iron deposits in the brain of migraine patients are still not completely clear. Migraine associated iron deposits are reported in the regions of the brain in which nociceptive signaling, important in migraine, occurs, so ferroptosis could induce changes in these pathways. At present, two aspects of ferroptosis in migraine may be considered: (1) it may be a consequence of iron deposits in specific regions of the brain of migraine patients, and (2) it may be an important element of increased oxidative stress in the migraine-affected brain, resulting from its enhanced energy demand and impaired pathways of energy production.

Iron in CNS is mainly stored in FT, common in glia and neurons, but can be also found in complexes with neuromelanin (NM), a catecholamine-derived pigment of the dopamine neurons of the substantia nigra and norepinephrine neurons of the locus coeruleus, which is an effective metal chelator (Zucca et al., 2017). Ferritin in the CNS occurs as a 24-subunit protein composed of two types of subunits—heavy (H-type FT) and light (L-type FT) chains, which are differentially expressed in different populations of neural cells (Arosio et al., 2017). The release of iron from its complex with FT is initiated by nuclear receptor coactivator 4 (NCOA4)-mediated autophagic degradation of FT, but such iron may induce cellular senescence and other pathological consequences unless exported or metabolized (Ito et al., 2021; Li H.-Y. et al., 2023).

**Figure 2:**
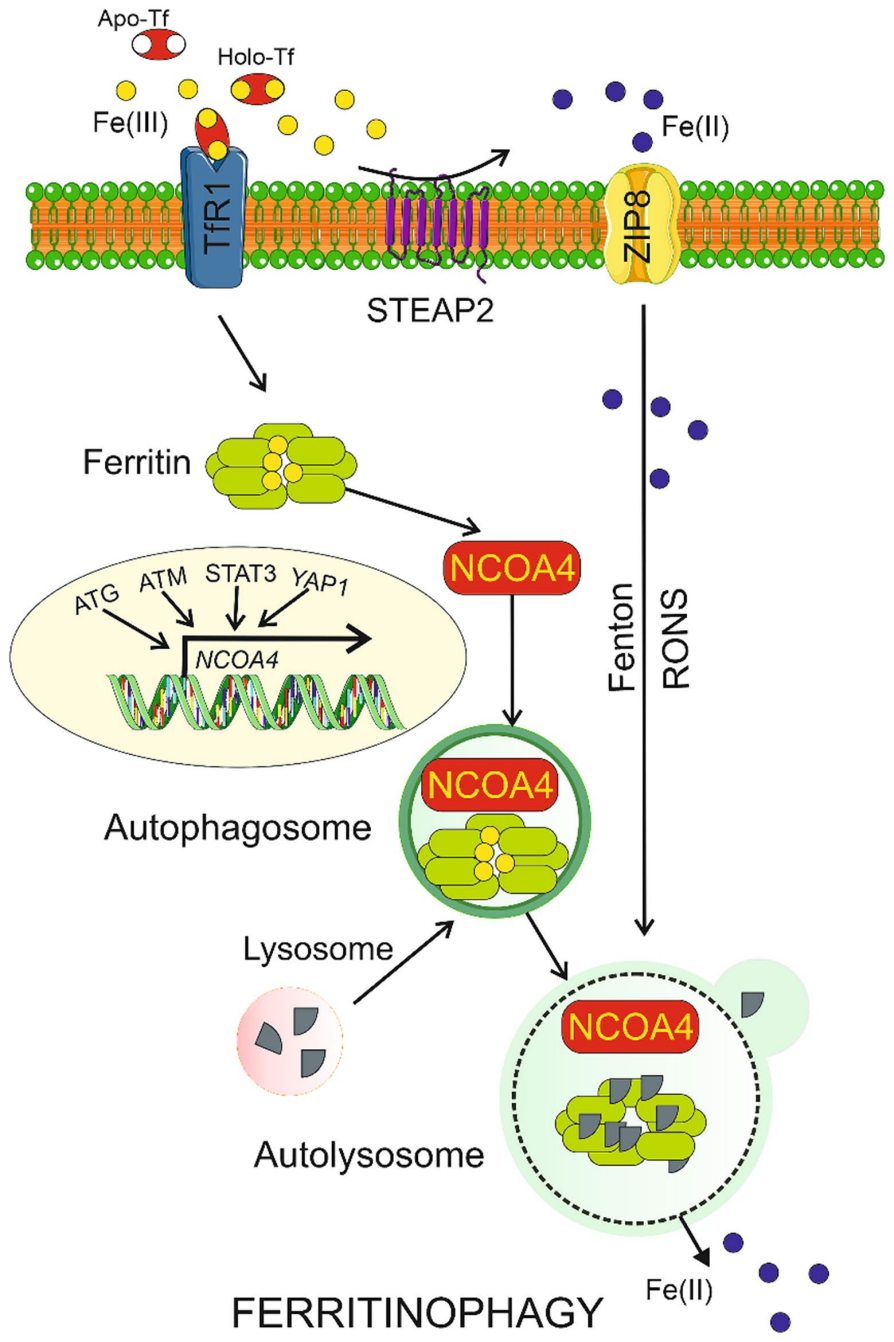
Ferritinophagy. Ferric ions (Fe(III)) are bound by apo-transferrin (Apo-Tf) to form holo-transferrin (Holo-Tf) that is transported by transferrin receptor 1 (TfR1) into the cell where they are bound by ferritin. Nuclear receptor coactivator 4 (NCOA4) may bind ferritin heavy chain and transport it along to the autophagosome, which is then fused with the lysosome to form autolysosome, in which ferritin is degraded in ferritinophagy and Fe(II) ions are released. Fe(III) can be reduced by the metalloreductase STEAP2 to Fe(II), which can directly enter the cell through divalent ion transporters represented here by ZIP8. Transcription of the NCOA4 gene is regulated by many factors including proteins encoded by the autophagy-related genes (ATG), ataxia-telangiectasia mutated (ATM), signal transducer and activator of transcription 3 (STAT3), and yes 1 associated transcriptional regulator (YAP1). Ferritinophagy may be also induced by reactive oxygen and nitrogen species (RONS) produced in the Fenton and Haber-Weiss reactions (Fenton) catalyzed by Fe(II). Parts of this figure were drawn by using pictures from Servier Medical Art. Servier Medical Art by Servier is licensed under a Creative Commons Attribution 3.0 Unported License (https://creativecommons.org/licenses/by/3.0/). Tables should be inserted at the end of the manuscript.

Several reports suggest a link between autophagy and ferroptosis underlined by the involvement of autophagy in the regulation of iron-dependent lipid peroxidation and RONS production during ferroptosis (Lee et al., 2023). We have recently argued that an interplay between degradative autophagy in neurons and secretory autophagy in the glia might protect the brain against prolonged consequences of migraine attacks (Fila et al., 2023b). Therefore, ferroptosis may be coupled with autophagy in migraine. However, at present it is a highly speculative hypothesis as neither autophagy nor ferroptosis was experimentally shown to be involved in migraine. Moreover, canonical autophagy is not specifically regulated by iron, but there is a kind of specific autophagy, ferritinophagy, which is involved in iron metabolism and ferroptosis (Figure 2). When the level of bioavailable iron is low, autophagy may cause nuclear receptor coactivator 4 (NCOA4)-mediated lysosomal degradation of ferritin and iron release (Mancias et al., 2014). Therefore, ferritinophagy may increase intracellular iron levels and RONS accumulation, resulting in ferroptosis (Gao et al., 2016). Ferritinophagy may contribute to ferroptosis but increased iron levels to stimulate ferroptosis can result from other than ferritinophagy effects (Sui et al., 2019). Moreover, ferritinophagy may be directly responsible for increased iron deposits in the brain. In summary, ferroptosis and ferritinophagy are important elements of iron metabolism. They should be considered as possible mechanisms mediating iron imbalance with a disease phenotype, as is the case of migraine.

## 5 Conclusions, perspectives, and outstanding questions

Increased iron deposits in certain brain areas have been associated with migraine occurrence and progression in several independent studies. Those studies underlined that the regions of increased iron deposition were in the brain areas important for migraine pathogenesis. However, no mechanistic explanations of how increased iron might affect those areas were provided. Iron-mediated production of RONS and subsequent damage to endothelial cells are usual endpoints in mechanistic considerations of consequences of increased iron deposits in migraine. We propose that ferroptosis, ferritinophagy, and their interplay may mechanistically link migraine with increased iron deposition in the brain.

One of the outstanding questions is why increased iron deposits are found only in certain areas of the brain of migraine patients. However, we cannot find a replicated study showing that certain structures in the brain are free of iron deposits in migraine. Therefore, there is a need to establish an atlas of the human brain with the areas affected and non-affected by iron deposition.

Considering the importance of iron metabolism, it is surprising that there are so few studies associating the concentration of iron in blood with migraine occurrence and characteristics. A clinical trial with dietary intervention with iron-rich and iron-poor foods in EM and CM migraine patients could shed light on the role of dietary iron as a trigger, a reliever, or a neutral substance in migraine. Increased iron deposits in the brain of the elderly may interfere with migraine-related iron deposits. The simplest way to solve this problem seems to be treating age-related iron as a confounding factor in the analysis of migraine-related iron, but this is not possible as the former cannot be distinguished from the latter. Therefore, studies on iron deposits in migraine patients must take care of as exactly as possible age-matched controls.

Autophagy raises an emerging interest in the study of the pathogenesis of various diseases, but there is a lack of experimental or clinical studies showing the involvement of autophagy in migraine pathogenesis. However, increased iron deposits in certain areas of the brain may be a source of many substrates for both degradative and secretory autophagy (Fila et al., 2023b). Iron deposited in the brain may stimulate ferritinophagy, which interplays with ferroptosis and may disturb nociceptive signaling important in migraine. Although there are some reports on animal models of migraine suggesting the occurrence of programmed cell deaths apoptosis and pyroptosis during the disease course, there is no experimental evidence on the involvement of cell death, including that induced by ferroptosis, in migraine pathogenesis. Again, there are no experimental data on the involvement of ferritinophagy and ferroptosis in migraine pathogenesis, but such involvement is likely due to the presence of increased iron deposits.

There are many other problems and outstanding questions on the role of iron in migraine, including the role of heme iron and ferritin-bound iron, reflecting the problem of systemic changes in migraine, but the results obtained so far point at iron as a potentially important player in migraine pathogenesis and justify further studies on this subject.

## Author contributions

MF: Conceptualization, Writing – original draft. LP: Writing – original draft. MD: Writing – original draft. JL-B: Writing – original draft. EP: Writing – original draft. JB: Conceptualization, Writing – original draft, Writing – review & editing.
